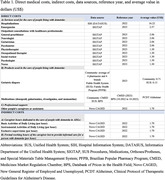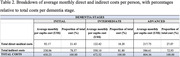# Direct and indirect costs of dementia in Brazil

**DOI:** 10.1002/alz.087782

**Published:** 2025-01-09

**Authors:** Fabiana A F da Mata, Ari Alex Ramos, Laiss Bertola, Thais Suarez, Cleusa P Ferri, Haliton Alves de Oliveira Júnior

**Affiliations:** ^1^ Hospital Alemão Oswaldo Cruz, São Paulo, São Paulo Brazil; ^2^ Universidade Federal de São Paulo (UNIFESP), São Paulo, São Paulo Brazil; ^3^ Universidade Federal de São Paulo (UNIFESP), São Paulo, São Paulo/SP Brazil

## Abstract

**Background:**

In Brazil, the dementia landscape is projected to intensify significantly, with the number of people living with dementia expected to more than triple by 2050. This anticipated surge underscores the urgency for health and social systems to strategize and prepare adequately. However, studies about costs related to dementia are very scarce in Brazil, making it difficult to understand better the financial impacts of the syndrome. Estimating the direct and indirect costs related to dementia, considering the reality of Brazil in its geographical diversity, is essential to understanding the reality of health resource allocation and, therefore, (re)direct resources to meet better the needs of people with dementia and their families. This study aims to estimate costs related to dementia in Brazil.

**Method:**

This study is part of the ReNaDe (National Report on Dementia) project, a domiciliary survey conducted with 140 dyads of people with dementia and their respective caregivers in 17 municipalities in Brazil. We used the cost of illness study methodology to estimate costs. We collected data from the ReNaDe interviews and national records and presented estimates from societal and the Brazilian Public Health Systems (SUS) perspectives. Our total costs encompassed direct medical costs (hospitalizations, outpatient visits, and others) and indirect costs (for instance, the monetary value of informal caregiving hours) (Table 1). We did not consider direct social costs, given limited or inexistent information about cost and services utilization by people with dementia in Brazil. We used the replacement approach to estimate indirect costs.

**Result:**

The monthly cost of dementia per individual increases with the syndrome’s progression (Table 2). Indirect costs, primarily associated with informal care provided by family or friends, constitute at least 73% of total expenses, irrespective of dementia stage and adopted perspective. For Brazil in 2019, dementia’s total annual cost was US$18 billion, with indirect costs comprising 78%.

**Conclusion:**

The average expenses per individual escalate with the advancement of dementia in Brazil. The pronounced prevalence of indirect costs accentuates the pivotal role that family caregivers assume in dementia care.